# A Novel 5-HT_1B_ Receptor Agonist of Herbal Compounds and One of the Therapeutic Uses for Alzheimer’s Disease

**DOI:** 10.3389/fphar.2021.735876

**Published:** 2021-09-06

**Authors:** Yang Yang, Lijing Zhang, Jiaojiao Yu, Zhaobin Ma, Moxiang Li, Jin Wang, Pengcheng Hu, Jia Zou, Xueying Liu, Ying Liu, Su An, Cheng Xiang, Xiaoxi Guo, Qian Hao, Tian-Rui Xu

**Affiliations:** Center for Pharmaceutical Sciences and Engineering, Faculty of Life Science and Technology, Kunming University of Science and Technology, Kunming, China

**Keywords:** 5-HT1B receptor, molecular docking, drug affinity responsive target stability, fluorescence resonance energy transfer, alzheimer’s disease, emodin-8-O-β-d-glucopyranoside

## Abstract

The serotonin receptor 5-HT_1B_ is widely expressed in the central nervous system and has been considered a drug target in a variety of cognitive and psychiatric disorders. The anti-inflammatory effects of 5-HT_1B_ agonists may present a promising approach for Alzheimer’s disease (AD) treatment. Herbal antidepressants used in the treatment of AD have shown functional overlap between the active compounds and 5-HT_1B_ receptor stimulation. Therefore, compounds in these medicinal plants that target and stimulate 5-HT_1B_ deserve careful study. Molecular docking, drug affinity responsive target stability, cellular thermal shift assay, fluorescence resonance energy transfer (FRET), and extracellular regulated protein kinases (ERK) 1/2 phosphorylation tests were used to identify emodin-8-O-β-d-glucopyranoside (EG), a compound from Chinese medicinal plants with cognitive deficit attenuating and antidepressant effects, as an agonist of 5-HT_1B_. EG selectively targeted 5-HT_1B_ and activated the 5-HT_1B_-induced signaling pathway. The activated 5-HT_1B_ pathway suppressed tumor necrosis factor (TNF)-α levels, thereby protecting neural cells against beta-amyloid (Aβ)-induced death. Moreover, the agonist activity of EG towards 5-HT_1B_ receptor, in FRET and ERK1/2 phosphorylation, was antagonized by SB 224289, a 5-HT_1B_ antagonist. In addition, EG relieved AD symptoms in transgenic worm models. These results suggested that 5-HT_1B_ receptor activation by EG positively affected Aβ-related inflammatory process regulation and neural death resistance, which were reversed by antagonist SB 224289. The active compounds such as EG might act as potential therapeutic agents through targeting and stimulating 5-HT_1B_ receptor for AD and other serotonin-related disorders. This study describes methods for identification of 5-HT_1B_ agonists from herbal compounds and for evaluating agonists with biological functions, providing preliminary information on medicinal herbal pharmacology.

## Introduction

The serotonergic system is important in regulating crucial processes in the central nervous system (CNS). These effects are mediated by serotonin receptors, such as the serotonin receptor subtype 1B (5-HT_1B_). These receptors, belonging to the G protein-coupled receptors (GPCRs) superfamily, are abundantly expressed in the CNS and constitute validated as well as putative drug targets in a variety of cognitive and psychiatric disorders, including depression and Alzheimer’s disease (AD), the most common incurable neurodegenerative disease (Monti and Jantos, 2008; Tiger et al., 2018; Gadgaard and Jensen, 2020).

Activation of the serotonergic system blocks the beta-amyloid (Aβ) oligomer-induced inflammatory response in AD (Ledo et al., 2016). Aβ-induced inflammatory response and neuronal death play important roles in AD pathology. Recent studies have identified inflammatory pathways as potential new drug targets for treating AD (Unzeta et al., 2016). Aβ oligomer-mediated activation of pro-inflammatory tumor necrosis factor (TNF)-α signaling in the brain has been reported as a cause of memory loss in mice (Lourenco et al., 2013). Activation of 5-HT_1B_ by its agonist sumatriptan, an anti-migraine agent, significantly diminishes the mRNA levels of TNF-α in rat nerve cells (Khalilzadeh et al., 2018). Moreover, it has been reported that the frontal and temporal cortices of AD patients show a reduction in 5-HT_1B/1D_ receptor levels (Garcia-Alloza et al., 2004). Therefore, the anti-inflammatory effects of 5-HT_1B_ agonists may present a promising approach in AD treatment.

Overall activation of 5-HT_1B_ in the CNS may have antidepressant effects (Sanchez et al., 2015). Several lines of evidence support the therapeutic use of herbal antidepressants in the treatment of AD (Jeon et al., 2019; Chen et al., 2020; Li et al., 2020), wherein various compounds present in the ingredients reduce neuronal inflammation and apoptosis. However, the targets of the herbal antidepressants and the interactions between the active compounds and their targets, which constitute the basic issue of medicinal herbal pharmacology, remain to be elucidated. The functional overlap between herbal antidepressants of traditional Chinese medicine and 5-HT_1B_ stimulation suggests the therapeutic potential of compounds from the ingredients of these medicinal plants, as they can target and stimulate 5-HT_1B_ for AD treatment.

In this study, the active compound emodin-8-O-β-d-glucopyranoside (EG), a component of several Chinese medicinal plants such as *Polygonum multiflorum* and *Rheum officinale* that contain herbal antidepressants that could be used in AD treatment (Lin et al., 2015; Jeon et al., 2019; Chen et al., 2020; Li et al., 2020), was identified as a ligand targeting the 5-HT_1B_ receptor. The role of serotonin-system-mediated neural protection was also investigated*.* The results support the importance of 5-HT_1B_ receptor as a therapeutic target and validate the potential use of 5-HT_1B_ agonists as therapeutic agents for serotonin system-related diseases such as AD.

## Materials and Methods

### Drugs, Chemicals, Reagents, and Other Materials

EG (cas 23,313–21‐5) was obtained from Weikeqi (Sichuan, China), CP‐94253 (4‐{5‐propoxypyrrolo [3,2‐b]pyridin‐3‐yl}‐1.2,3,6‐tetrahydropyridine, catalog # SML0588), DOI (1‐(4‐iodo‐2,5‐dimethoxyphenyl)propan‐2‐amine, catalog #D101), dopamine (4‐(2‐Aminoethyl)benzene‐1,2‐diol, catalog #H8502), and caffeine from Sigma‐Aldrich, ramelteon (N‐[2‐[(8S)‐2.6,7,8‐tetrahydro‐1H‐cyclopenta [e][1]benzoxol‐8‐yl]ethyl]propanamide) from MedChemExpress (catalog # HYA0014), and dihydroergotamine ((2,4, 7R)‐N‐[(1S,2S,4R, 7S)‐7‐benzyl‐2‐hydroxy‐4‐methyl‐5,8‐dioxo‐3‐oxa‐6,9‐diazatricyclo[7.3.0.0  ^∧^  {2.6}]dodecan‐4‐yl]‐6‐methyl‐6,11‐diazatetracyclo [7.6.1.0^∧^{2.7}.0 ^∧^ {12.16}]hexadeca‐1 (16),9,12,14‐tetraene‐4‐carboxamide)and SB 224289 ([4‐[2‐methyl‐4‐(5‐methyl‐1,2,4‐oxadiazol‐3‐yl)phenyl]phenyl]‐(1′‐methylspiro [6,7‐dihydro‐2H‐furo [2,3‐f]indole‐3.4′‐piperidine]‐5‐yl)methanone) from APEx BIO (catalog #B3459 and B6641, respectively). OxB (RSGPPGLQGRAQRLLQASGNHAAGILTM‐NH2), Aβ40 (rat:DAEFGHDSGFEVRHQKLVFFAEDVGSNKGAIIGLMVGGVV; human: DAEFRHDSGYEVHHQKLVFFAEDVGSNKGAIIGLMVGGVV) and Aβ42 (rat: DAEFGHDSGFEVRHQKLVFFAEDVGSNKGAIIGLMVGGVVIA; human: DAEFRHDSGYEVHHQKLVFFAEDVGSNKGAIIGLMVGGVVIA) were synthesized by Shangting (Shanghai,China). The Flp‐In™ T‐REx™ 293 cells, Lipofectamine®2000 transfection reagent, and cell culture materials were from Invitrogen (Thermo Fisher Scientific Inc., United States). The anti‐VSV‐G antibody (catalog #V5507) was obtained from Sigma‐Aldrich, and the anti‐ERK1/2‐MAP kinase (catalog # 9102S) and anti‐phospho‐ERK1/2‐MAP kinase (catalog # 9101S) antibodies were from Cell Signaling Technology (Nottingham,United Kingdom).

### DNA Constructs

The VSV-G-mGluR5-5-HT_1B,_ VSV-G-mGluR5-5HT_2A_, D_2_, MT_2_, and OX_2_ constructs were established by inserting human 5-HT_1B_ cDNA into a pcDNA5/FRT/TO vector (Invitrogen) using the In-Fusion® PCR Cloning System (Clontech, United States) as previously described (Xu et al., 2012). Constructs were introduced into HEK293T cell lines for preliminary transient transfection studies and then into Flp-In™ T-REx™ 293 cells to generate inducible stable cell lines (Ward et al., 2011).

### Generation and Maintenance of Stable Flp-In™ T-REx™ 293 Cells

The cells were co-transfected with the pOG44 plasmid and the desired protein cDNA in pcDNA5/FRT/TO in a 9:1 ratio using Lipofectamine®2000 to generate Flp-In™ T-REx™ 293 cells that can inducibly express the indicated constructs (Ward et al., 2011). After 48 h, the medium was supplemented with 200 ng ml^−1^ hygromycin to select stable transfected cells. The cell pools were tested for inducible expression by adding 100 ng ml^−1^ doxycycline for 12 h, followed by western blot analysis for phosphorylation of extracellular regulated ERKs corresponding to GPCR activation and VSV-G protein expression.

### Cell Culture and Transfection

HEK293T cells were maintained in Dulbecco’s modified Eagle’s medium supplemented with 0.292 g⋅ L^−1^ l-glutamine, whereas PC12 and SH-SY5Y cells were maintained in PRIM 1640 medium and 10% (v/v) fetal bovine serum (FBS) at 37°C in a 5% CO_2_ humidified atmosphere. The cells were transfected with the indicated constructs using Lipofectamine®2000.

### Molecular Docking Process

The structure of 5-HT_1B_, access number 5V54 (Name: Crystal structure of 5-HT_1B_ receptor in complex with methiothepin), was obtained from the PDB database (http://www.rcsb.org/pdb/home/home.do). The 3D structures and properties of CP-94253 and EG were obtained from the PubChem database (https://pubchem.ncbi.nlm.nih.gov). The free molecular docking software AutoDock Tools-1.5.6 was employed, and the Autodock 4 (Department of Molecular Biology of Scripps Research Institute, La Jolla, CA and Department of Cognitive Science of University of California, San Diego, La Jolla, CA) was used for flexible docking, as previously reviewed and discussed (Beuming et al., 2015; Kontoyianni, 2017).

### Cell Lysates and Western Blotting

Cells were washed once in cold phosphate-buffered saline (PBS) (120 mM NaCl, 25 mM KCl, 10 mM Na_2_HPO_4_, and 3 mM KH_2_PO_4_, pH 7.4) and harvested with ice-cold radioimmunoprecipitation assay (RIPA) buffer (50 mM HEPES, 150 mM NaCl, 1% Triton X-100, and 0.5% sodium deoxycholate, 10 mM NaF, 5 mM EDTA, 10 mM NaH_2_PO_4_, and 5% ethylene glycol pH 7.4) supplemented with a protease inhibitor cocktail, as previously described. After heating the samples at 37°C for 5 min, the cell lysates were subjected to sodium dodecyl sulfate-polyacrylamide gel electrophoresis. The proteins were electrophoretically transferred onto a nitrocellulose membrane, which was then blocked (5% fat-free milk powder in PBS containing 0.1% Tween-20) at room temperature on a rotating shaker for 1 h, washed twice in tris-buffered saline (TBS, pH 7.4) containing 0.1% Tween 20, and incubated with the appropriate primary antibody for 2 h, followed by incubation with a secondary antibody for 1 h at room temperature. Subsequently, the proteins of interest were visualized using the ECL Chemiluminescence System (Santa Cruz Biotechnology) according to the manufacturer’s instructions. Protein levels were quantified by band intensity using the Quantity One 1D Analysis Software.

### DARTS and CETSA

Intact Flp-In™ T-REx™ 293 cells induced to express mGluR5-VSV-G-5-HT_1B_ were treated with EG, dihydroergotamine, CP-94253, or the vehicle at the indicated concentrations and used for subsequent studies. In the DARTS experiment, cell lysates were subjected to pronase digestion at room temperature for 3 min, then loaded for western blotting to detect the 5-HT_1B_ protein. In the CETSA experiment, the cells were scraped off and lysed via alternate freezing and thawing thrice with liquid nitrogen, then heated to varying temperatures for 5 min. The 5-HT_1B_ protein was detected with western blotting. In both experiments, GAPDH was used as the loading control for quantification.

### FRET Experiments

The 5-HT_1B_ FRET sensor-expressing cells were placed in a microscope chamber containing physiological HEPES-buffered saline solution (130 mM NaCl, 5 mM KCl, 1 mM CaCl_2_, 1 mM MgCl_2_, 20 mM HEPES, and 10 mM d-glucose, pH 7.4). The cells were then imaged using an inverted Nikon TE2000-E microscope (Nikon Instruments) equipped with a 40× (numerical aperture = 1.3) oil immersion Fluor lens, as previously described (Xu et al., 2012). The monochromator was set at 427 nm/bandwidth (BW) 5 and 504 nm/BW 5 nm to visualize the surface located cyan fluorescent protein (CFP) and FlAsH separately. FRET and donor emission images were recorded simultaneously using a Quadview 2 (QV2) image splitting device (Photometrics, United Kingdom), coupled to a CoolSnap-HQ2 camera connected to the microscope. The FRET and donor signals were detected simultaneously using the following Chroma (Brattleboro, VT) ET dichroic and emitter series mounted in the QV2 cube: ET t505LPXR dichroic, ET535/30 nm, and ET 632/60 nm. Using the streaming capability of the multiple dimensional wavelength acquisition modules of MetaMorph, the ligand-induced changes in intramolecular FRET were recorded directly in the computer’s hard drive at 40 ms intervals during excitation with 427 nm light. The Cool Snap-HQ2 camera was operated in the 14-bit mode, and exposure time, 8 × 8 binning, and camera gain, were kept constant for all streaming experiments. Computerized control of all electronic hardware and camera streaming acquisition was achieved using the MetaMorph software (version 7.7.5 Molecular Devices, Sunnydale, CA). A peristaltic pump, operated at a 5 ml/min flow rate, was used to rapidly add or remove test ligands to or from the imaging chamber. The ratiometric quantification of intramolecular charge changes was then calculated as the average 535 nm emission intensity divided by the average 470 nm emission intensity. The FRET ratiometric was set to 1.0 at the onset of each experiment and plotted over time.

### ERK1/2 MAP Kinase Phosphorylation

Cells stably expressing inducible GPCRs were placed in 6-well plates and allowed to grow overnight. Expression of the constructs was induced by adding 10 ng ml^−1^ doxycycline for 12 h and rendered quiescent via serum starvation for 12 h, then stimulated for 5 min by the indicated agonist using FBS as the positive control and deionized water as the negative control. Cells were then placed on ice and harvested with ice-cold RIPA buffer. Phosphorylation of ERK1/2 MAP kinases was detected via western blotting using a phospho-ERK1/2-specific antibody. The nitrocellulose membranes were subsequently stripped of immunoglobulins and re-probed using an anti-ERK1/2 antibody to assess the protein loading equivalence.

### Quantitative RT-PCR

Total RNA was isolated from PC12 or worm model cells treated as previously indicated, using TRIzol (Ambion Life Technologies), and the mRNA was reversed-transcribed using the RevertAid First Strand cDNA Synthesis Kit (Thermo Fisher, catalog #K1621) according to the manufacturer’s instructions. The primer sequences for quantitative PCR were as follows: TNF-α F (5′-CCC​TCA​CAC​TCA​GAT​CAT​CTT​CT-3′), TNF-α R (3′-GCT​ACG​ACG​TGG​GCT​ACA​G-5′), β-Actin F (5′-GAT​TAC​TGC​TCT​GGC​TCC​TAG​C-3′), and β-Actin R (3′-GAC​TCA​TCG​TAC​TCC​TGC​TTG​C-5′). Previously described primers were used for the serotonin receptor subtypes (Koizumi and Nakajima, 2014) and the worm model (Bellier et al., 2009; Sahu et al., 2012). Three independent experiments were performed on triplicate samples. PCR amplification was performed using the SYBR Green PCR Master Mix Kit (Thermo Fisher). All quantifications were normalized to *GAPDH* levels.

### CCK-8, MTT, and LDH Assays

The PC12 cells were seeded in a 96-well plate at a 5 × 10^3^ density; 12 h after seeding, CP-94253 or EG was added with or without SB 224289. After 24 h of seeding, the amyloid peptides were added, and after 18 h, the cells were subjected to CCK-8 (catalog #C0037, Beyotime, China), MTT (catalog #M1025, Solarbio, Beijing, China), and LDH (catalog # BC0680, Solarbio, Beijing, China) assays following the manufacturers’ instructions.

### *C. Elegans* Assay for AD Protective Compounds

The *C. elegans* CL14176 strain was synchronized to the L1 phase and allocated to 160 μL S media with the appropriate treatment drug concentrations. The worms were cultured at 16°C for 24 h and transferred to 25°C, at which point they were activated to express amyloid peptides that would result in paralysis (Dostal and Link, 2010). At 38 h, the worms were counted for the paralysis phenotype. Each group contained approximately 60 worms. The positive control group was set by adding 6.27 mM caffeine.

### Statistical Analysis

Variables were compared between non-treated and treated groups using Student’s t-tests. Statistical differences in protein expression, cell viability, and relative mRNA levels between two groups were analyzed using a One-way Analysis of variance (ANOVA). Data were analyzed using GraphPad Prism 6.01 software (GraphPad Software, La Jolla, CA, United States). Data are expressed as mean ± S.D. of values from at least three independent experiments. A value of *p* < 0.05 was considered statistically significant.

## Results

### EG Targets 5-HT_1B_ Receptor

**FIGURE 1:**
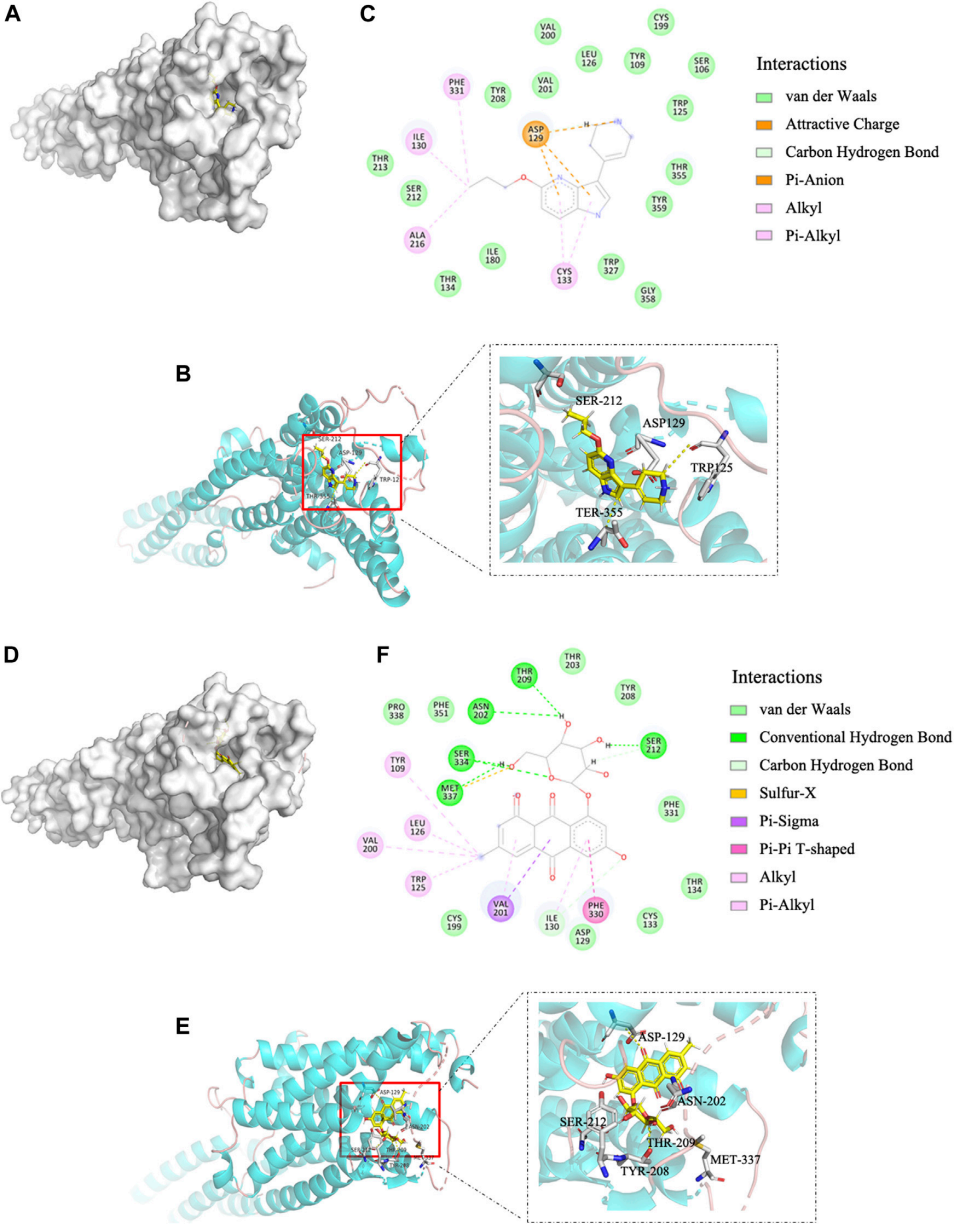
EG is a 5-HT_1B_ ligand in molecular docking compared with agonist CP-94253. **(A)** Electrostatic surface model. **(B)** Schematic representation of crystal structure. **(C)** Magnified 2D-view of protein-ligand CP-94253 docking into its 5-HT_1B_ interacting pocket. **(D−F)** Corresponding representations of EG docking into the same 5-HT_1B_ interacting pocket as CP-94253. EG: emodin-8-O-β-d-glucopyranoside; 5-HT_1B_: serotonin receptor 1B; CP-94253: 4-{5-propoxypyrrolo [3,2-b]pyridin-3-yl}-1,2,3,6-tetrahydropyridine.

Molecular docking is widely used to identify specific molecular targets, such as GPCRs (Beuming et al., 2015; Kontoyianni, 2017). We used this technique to determine whether EG binds to the human GPCR 5-HT_1B_. The binding affinity of EG to 5-HT_1B_ was similar to that of the agonist CP-94253, as the binding energies were −8.09 and −8.48 kcal mol^−1^, respectively. EG (Figures 1D,E) docked into the same 5-HT_1B_ pocket as CP-94253 (Figures 1A,B). In addition, EG (Figure 1F) interacted with 5-HT_1B_ similarly to the agonist CP-94253 (Figure 1C). The specific interaction parameters are shown in Supplementary Figure S1. The results suggested that EG might target 5-HT_1B_ and act similarly to CP-94253 as a ligand of this receptor.

**FIGURE 2:**
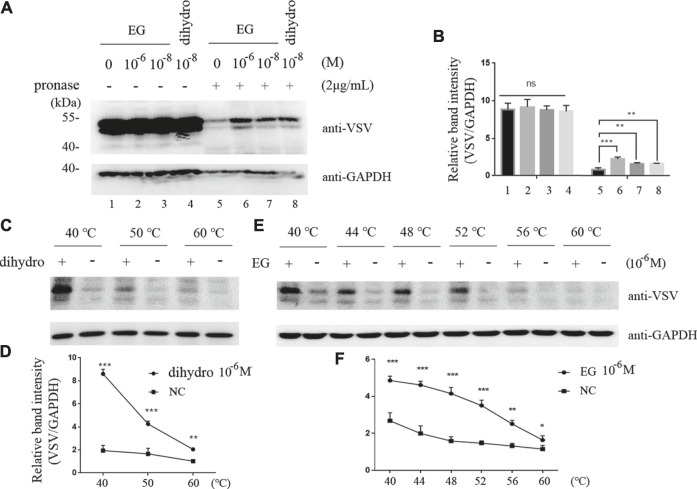
EG interaction with 5-HT_1B_ in DARTS and CETSA experiments. **(A,B)** Intact Flp-In™ T-REx™ 293 cells stably induced to express mGluR5-VSV-G-5-HT_1B_ were treated with EG or dihydro (dihydroergotamine, a 5-HT_1B_ agonist) at indicated concentrations and lysates were subjected to pronase (2 μg/ml) digestion. 5-HT_1B_ protein with EG and dihydro protection in pronase digestion detected by western blotting and enrichment of 5-HT_1B_ protein in pronase-induced depletion quantified by relative band intensity compared with GAPDH. **(C,D)** Intact Flp-In™ T-REx™ 293 cells of stably expressing mGluR5-VSV-G-5-HT_1B_ treated with dihydro compared to control cells. The harvested cells were lysed three times by alternate freezing and thawing with liquid nitrogen before heating to the indicated temperatures. The 5-HT_1B_ engagement with dihydro was detected by western blotting and quantified by relative band intensity compared with GAPDH. The corresponding 5-HT_1B_ thermodynamic stabilization curves distinguish the treated from non-treated cells. **(E,F)** Stable cells expressing mGluR5-VSV-G-5-HT_1B_ treated with EG compared to control cells. The cells were alternately frozen and thawed three times followed by heating to the indicated temperatures, and the 5-HT_1B_ engagement with EG was detected by western blotting. The corresponding 5-HT_1B_ thermodynamic stabilization curves distinguished the treated from non-treated cells. The mean values (±S.D.) of three independent experiments are shown. An asterisk indicates statistical significance (*p < 0.05, **p < 0.001, ***p<<0.001).

To verify the interaction of EG with the 5-HT_1B_ receptor, we applied DARTS and CETSA, previously reported methods (Chang et al., 2016), for identifying target proteins of natural products without chemical modifications. Unlike that with the single target protein, the conformation and stabilization of the compound-target complex are altered in either DARTS or CETSA, and the interaction between the compound and target protein can be visualized via western blotting (Lomenick et al., 2009; Molina et al., 2013; Chang et al., 2016). In this study, DARTS was performed on the whole-cell lysate of cells stably induced to express 5-HT_1B_. The proteins were digested with pronase, whereas EG, CP-94253, and dihydroergotamine (a 5-HT_1B_ agonist, FDA-approved for migraine) were used to prevent target depletion. The 5-HT_1B_ protein in the band protected by EG (10^−6^ M) was enriched approximately 2-fold compared with that in the vehicle, and EG showed a similar protective effect with dihydroergotamine at a low concentration (10^−8^ M). Glyceraldehyde 3-phosphate dehydrogenase (GAPDH) was resistant to pronase under this condition and served as a loading indicator (Figures 2A,B). The 5-HT_1B_ protein protected by EG, compared with CP-94253, from digestion is shown in Supplementary Figure S2A. CETSA was performed using intact cells induced to express 5-HT_1B_. The dihydroergotamine- or vehicle-treated cells were heated to varying temperatures as indicated. The 5-HT_1B_ protein in the soluble fractions was separated from the precipitated destabilized protein and detected with western blotting (Figure 2C). Figure 2D shows ligand-target interaction plotted against temperature to display the shifts. The results revealed a physical interaction between the agonist dihydroergotamine and its target protein 5-HT_1B_ in intact cells. Additionally, EG-induced thermodynamic stabilization of 5-HT_1B_ was observed (Figures 2E,F). The 5-HT_1B_ protein thermodynamically stabilized by EG, compared with CP-94253, is shown in Supplementary Figure S2B.

**FIGURE 3:**
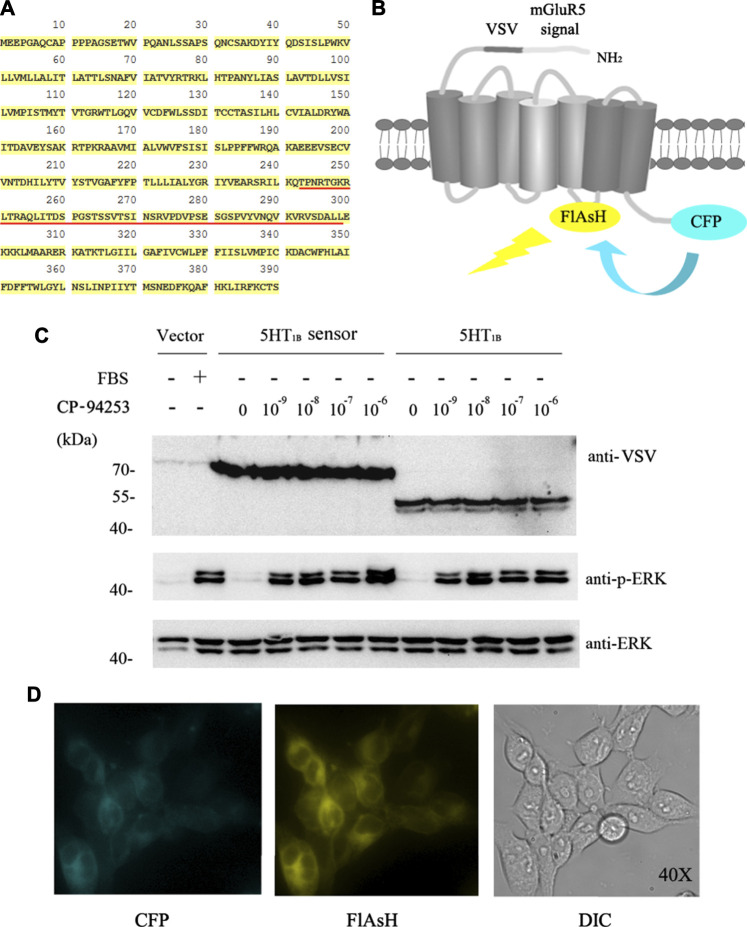
5-HT_1B_ FRET sensor construction. **(A)** Primary human 5-HT_1B_-receptor amino acid sequence. FlAsH motif replaces FRET sensor sequence (underlined in red). **(B)** N terminus modification of human 5-HT_1B_ receptor by adding a leader sequence from mGluR5 followed by the VSV-G peptide sequence, while CFP was added at C terminus. FlAsH motif sequence FLNCCPGCCMEP introduced into third intracellular loop-linking transmembrane domains V and VI. Energy transfer from CFP to FlAsH and subsequent output is illustrated. **(C)** Stable cell lines harboring inducible 5-HT_1B_ FRET sensor, VSV-G-mGluR5-5-HT_1B_, and corresponding vector constructs induced by 10 ng ml^−1^ doxycycline for 12 h, followed by 12 h of serum starvation quiescence, and 5 min stimulation by graded concentrations (10^−9^ to 10^−6^ M) of the 5-HT_1B_ selective agonist CP-94253, using FBS as the positive control and deionized water as negative control in vector construct cells. Samples were then subjected to western blotting. The expression of 5-HT_1B_ was determined by its fusion protein tag using VSV antibody. Phosphorylation of ERK 1/2 MAP kinases was detected by a phospho-ERK1/2-specific antibody (anti-p-ERK), and the anti-ERK1/2 antibody was used to assess the protein loading equivalence. **(D)** 5-HT_1B_ FRET sensor was cloned into the inducible Flp-In™ T-REx™ locus of Flp-In™ T-REx™ 293 cells, and expression was induced by doxycycline. Imaging of CFP **(left)**, labeled FlAsH **(middle)**, and light field **(right)** demonstrated effective delivery of this sensor to the cell surface. FRET: fluorescence resonance energy transfer; VSV: vesicular stomatitis virus; FlAsH: fluorescein arsenical hairpin binder via tetra-cysteine; ERK: extracellular regulated protein kinases; CFP: cyan fluorescent protein; VSV: vesicular stomatitis virus; EG: emodin-8-O-β-d-glucopyranoside; CP-94253: 4-{5-propoxypyrrolo [3,2-b] pyridin-3-yl}-1.2,3,6-tetrahydropyridine; 5-HT_1B_: serotonin receptor 1B; FBS: fetal bovine serum; DIC: differential interference contrast; mGluR5: metabotropic glutamate receptor 5.

Intramolecular FRET sensors have been used as real-time optical tools for GPCR ligand binding (Xu et al., 2012; Lohse et al., 2008). We successfully produced effective 5-HT_1B_ sensor constructs using previously described methods (Xu et al., 2012). The full-length human 5-HT_1B_ receptor was added to CFP in-frame with the C-terminal tail (Figures 3A,B), and the 12-amino acid sequence (FLNCCPGCCMEP) containing the fluorescein arsenical hairpin binder via tetra-cysteine (FlAsH) labeling sequence (CCPGCC) was inserted into the third intracellular loop of the receptor. In addition, it contained the VSV-G peptide epitope within the extracellular N-terminal domain (Figure 3B). Functionality of the 5-HT_1B_ sensor has been previously assessed in a study on ERK1/2 MAP kinase phosphorylation (p-ERK) (Xu et al., 2012). The full-length coding sequence of the human 5-HT_1B_ receptor without alterations to the receptor’s intracellular segments was also cloned into the pcDNA5/FRT/TO vector described previously (Xu et al., 2012) and in the Methods section. Then, these constructs were transfected into Flp-In™ T-REx™ 293 cells to generate stable cell lines. In these cells, the doxycycline-induced expression of either the 5-HT_1B_ sensor or the full-length human 5-HT_1B_ was examined using an anti-VSV antibody. The 5-HT_1B_ sensor performed effectively in response to the selective 5-HT_1B_ agonist CP-94253, compared with the full-length human 5-HT_1B_, at the p-ERK level (Figure 3C). The 5-HT_1B_ sensor was effectively delivered to the cell surface, on which its normal functions are based (Figure 3D). These results showed that the sensor was functionally similar and virtually identical to the wild-type receptor.

**FIGURE 4:**
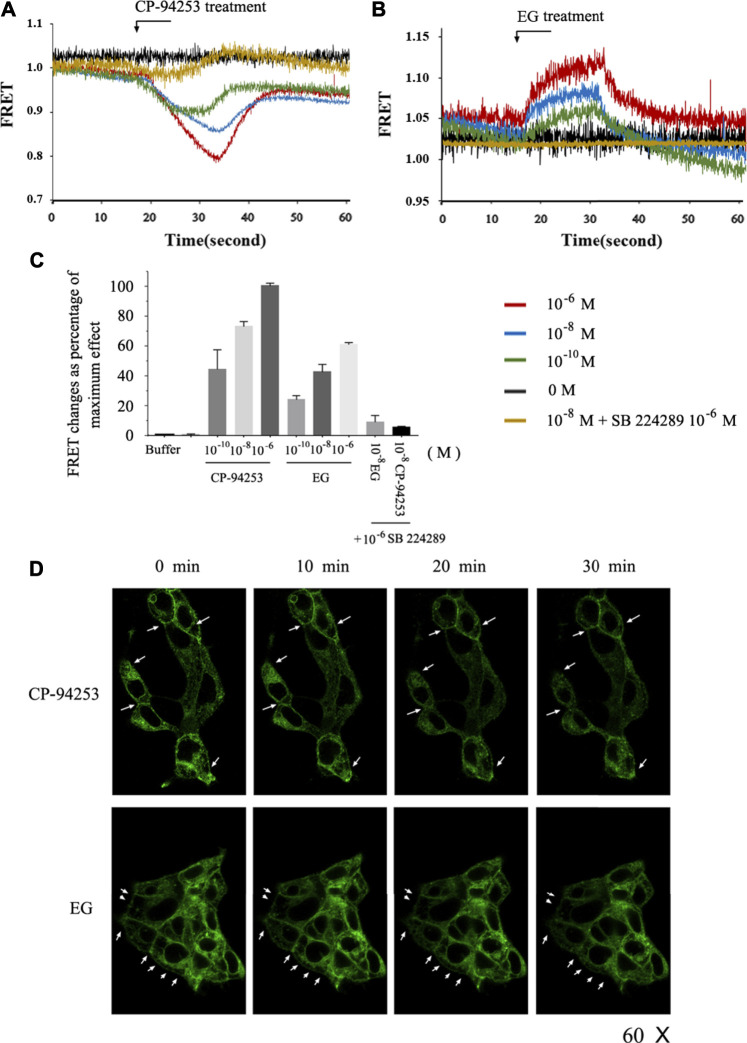
Intramolecular FRET sensor imaging of EG as a 5-HT_1B_ agonist. **(A,B)** Flp-In™ T-REx™ 293 cells induced to express mGluR5-VSV-G-5-HT_1B_-FlAsH-CFP shown in FRET imaging. CP-94253** (A)** or EG **(B)** at three concentrations (10^−10^, 10^−8^ and 10^−6^ M) added at the indicated times and removed after 15 s. FRET signal was monitored over 60 s. **(C)** Changes in normalized FRET signals due to ligand addition and effect of 10^−6^ M CP-94253 defined as 100% and vehicle as 0%. 5-HT_1B_ receptor antagonist SB 224289 did not modulate mGluR5-VSV-G-5-HT_1B_-FlAsH-CFP FRET signals, but co-addition with CP-94253 or with EG blocked agonists’ effects. Studies were quantified, and data are represented as means ± S.E., n = 6. **(D)** Flp-In™ T-REx™ 293 cells induced to express mGluR5-VSV-G-5-HT_1B_-FlAsH-CFP imaged to detect CFP following addition of CP-94253 or EG (10^−7^ M). CP-94253 or EG-induced internalization, reducing the cell surface mGluR5-VSV-G-5-HT_1B_-FlAsH-CFP in a time-dependent fashion. Representative examples of n = 3 independent experiments are shown. FRET: fluorescence resonance energy transfer; FlAsH: fluorescein arsenical hairpin binder via tetra-cysteine; CFP: cyan fluorescent protein; VSV: vesicular stomatitis virus; EG: emodin-8-O-β-d-glucopyranoside; CP-94253: 4-{5-propoxypyrrolo [3,2-b]pyridin-3-yl}-1.2,3,6-tetrahydropyridine; SB 224289 [4-[2-methyl-4-(5-methyl-1,2,4-oxadiazol-3-yl)phenyl]phenyl]-(1′-methylspiro [6,7-dihydro-2H-furo [2,3-f]indole-3.4′-piperidine]-5-yl) methanone; 5-HT_1B_: serotonin receptor 1B; mGluR5: metabotropic glutamate receptor 5; FlAsH: fluorescein arsenical hairpin binder via tetra-cysteine.

To study the effects of EG on the 5-HT_1B_ receptor, we imaged the intramolecular rearrangement of the 5-HT_1B_ sensor in response to EG, compared with CP-94253, by measuring emission from CFP and FlAsH. The normalized basal FRET signal in cells induced to express the 5-HT_1B_ sensor decreased immediately upon the addition of CP-94253, then rapidly restored agonist withdrawal in a concentration-dependent manner. In contrast, addition of the 5-HT_1B_ antagonist SB 224289 (10^−6^ M) did not produce a significant alteration in sensor response beyond the vehicle effect, and, as anticipated, blocked the effect of CP-94253 (Figure 4A). This was similar to the results obtained using EG, although the FRET signal changed in the opposite direction to that of CP-94253 (Figure 4B). Moreover, the variability of FRET changes from EG was approximately 60% of CP-94253 (Figure 4C), illustrating that structure changes in the receptor triggered by binding of CP-94253 and EG were distinct in amplitude and distance, at least between the third intracellular loop and C-terminal, where CFP and FlAsH are located, respectively. 5-HT_1B_ has been previously shown to be internalized from the cell surface via agonist binding in a time-dependent manner (Janoshazi et al., 2007). Our findings revealed that EG induced 5-HT_1B_ internalization, similarly to CP-94253 (Figure 4D). These results demonstrated that EG could be a 5-HT_1B_ agonist.

### EG Showed Selective Agonist Activity Toward 5-HT_1B_ Receptor Rather Than Other GPCRs

**FIGURE 5:**
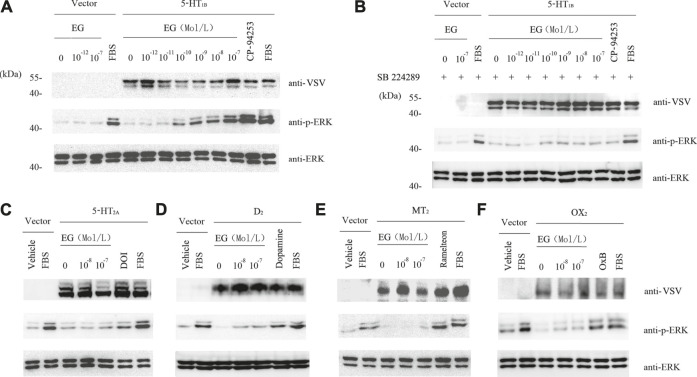
EG selectively activates 5-HT_1B_ signaling. **(A)** Stable cell lines harboring inducible VSV-G-mGluR5-5HT_1B_ and corresponding vector constructs induced by 10 ng ml^−1^ doxycycline for 12 h, and another 12 h of serum starvation for quiescence, then stimulated for 5 min by graded concentrations of EG (10^−12^ to 10^−7^ M in 5-HT_1B_ cells, 10^−12^ to 10^−7^ M in vector cells), and the 5-HT_1B_ selective agonist CP-94253 at 10^−7^ M, using FBS as positive control and deionized water as negative control. Samples were then subjected to western blotting. The expression of 5-HT_1B_, phosphorylation of ERK1/2 MAP kinases and loading control of ERK1/2 were determined by indicated antibodies described above in legend of Figure 3. **(B)** Stable cell lines and the inducing and starvation treatments were similar, as described in A above. Cells were pre-incubated with the 5-HT_1B_ selective antagonist SB 224289 at 10^−7^ M for 20 min, following stimulation and western blotting. **(C−F)** Stable cell lines harboring inducible VSV-G-mGluR5-5HT_2A_ **(C)**, VSV-G-mGluR5-D_2_ **(D)**, VSV-G-mGluR5-MT_2_ **(E)**, and VSV-G-mGluR5-OX_2_ **(F)** and the corresponding vector constructs treated similar to A and the effects of EG on the indicated GPCR mediated pathways determined by phosphorylation of ERK1/2 MAP kinases. The induced and quiescent stable cell lines were stimulated for 5 min by graded concentrations of EG (10^−8^ and 10^−7^ M), and the selective agonis at 10^−7^ M, that were DOI, dopamine, ramelteon and OxB, respectively corresponding to 5HT_2A_, D_2_, MT_2_ and OX_2_ cells, using FBS as positive control and deionized water as negative control. EG: emodin-8-O-β-d-glucopyranoside; FBS: fetal bovine serum; ERK: extracellular regulated protein kinases; CFP: cyan fluorescent protein; 5-HT_1B_: serotonin receptor 1B; 5-HT_2A_: serotonin receptor 2A; D_2_: dopamine receptor 2; MT_2_: melatonin receptor 2; OX_2_: orexin receptor 2; VSV: vesicular stomatitis virus; mGluR5: metabotropic glutamate receptor 5; FBS: fetal bovine serum; DOI (1-(4-iodo-2,5-dimethoxyphenyl) propan-2-amine; dopamine: 4-(2-Aminoethyl) benzene-1,2-diol; ramelteon (N-[2-[(8S)-2.6,7,8-tetrahydro-1H-cyclopenta [e] (Gadgaard and Jensen, 2020) benzoxol-8-yl]ethyl]propanamide).

To further study the EG target, we used stable cell lines that were induced to overexpress serotonergic-related GPCRs, including the serotonin receptors 1B and 2A (5-HT_1B_ and 5-HT_2A_), dopamine receptor 2 (D_2_), melatonin receptor 2 (MT_2_), and orexin receptor 2 (OX_2_). In the stable cell lines that over-expressed 5-HT_1B_, EG promoted the phosphorylation of ERK1/2 MAP kinases in a concentration-dependent manner. This activation through the 5-HT_1B_ receptor-mediated pathway was similar to the 5-HT_1B_ selective agonist, i.e., the positive control CP-94253 (Figure 5A). In addition, the 5-HT_1B_ selective antagonist SB224289 at 10^−7^ M inhibited the effect of the concentration-dependent promotion of the ERK1/2 MAP kinase phosphorylation by EG and CP-94253 activity (Figure 5B). However, no response to the EG of other 5-HT-related GPCRs activities, such as 5-HT_2A_, D_2_, MT_2,_ or OX_2,_ was observed (Figures 5C–F). These results showed that EG exhibited agonistic activity toward 5-HT_1B_ and could selectively activate the 5-HT_1B_-mediated signaling pathway.

### EG Alleviates Aβ-Induced Cell Mortality

**FIGURE 6:**
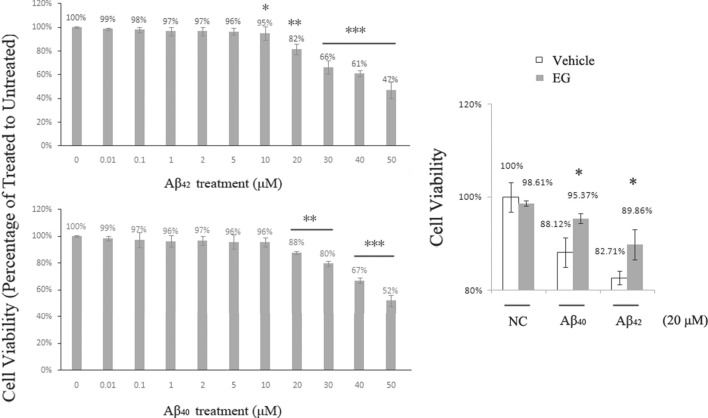
Neuronal death induced by Aβ peptides was alleviated by EG. **(A)** PC12 cells treated with amyloid peptides Aβ_42_ **(upper)** and Aβ_40_ **(lower)** for 24 h, at gradient concentrations from 0.01 to 50 μM, followed by Cell Counting Kit-8 (CCK-8) assay for viability. **(B)** PC12 cells pre-treated with (gray bar) or without (white bar) EG at 10^−7^ M for 18 h, then further subjected to 20 μM final concentration of amyloid peptides Aβ_40_ and Aβ_42_ for 24 h followed by CCK-8 assay for viability. The mean values (±S.D.) of three independent experiments are shown. An asterisk indicates statistical significance (*p < 0.05, **p < 0.001, ***p<<0.001). EG: emodin-8-O-β-d-glucopyranoside; NC: negative control.

Aβ-induced neuronal toxicity is an important pathology in AD, and there are two principal variants of amyloid peptides in humans, of which Aβ_42_ is more toxic than Aβ_40_ (Hamley, 2012). EG and its derivatives exhibit neural protection activity and can be used in AD treatment (Lin et al., 2015). We applied Aβ_40_ and Aβ_42_ to PC12 (with rat Aβ) and SH-SY5Y (with human Aβ) cell lines, respectively. The cells were subsequently assessed using the Cell Counting Kit-8 (CCK-8) for viability assay. Cell survival decreased with increased Aβ concentration in both PC12 (Figure 6A) and SH-SY5Y cells (Supplementary Figure S3). No significant differences in viability were observed in the treatments with <5 μM Aβ_42_ or <10 μM Aβ_40_. PC12 cell death was observed at 10 μM Aβ_42_ and reached approximately 18% at 20 μM, whereas cell death in the Aβ_40_ treatment group began at 20 μM. At 50 μM, 47% of the Aβ_42_-treated and 52% of the Aβ_40_-treated cells survived. These results suggest that Aβ peptides dose-dependently induce neuronal toxicity, and support the hypothesis that Aβ_42_ is more toxic than Aβ_40_. To determine the effects of EG on neural cells, we pre-treated PC12 cells with or without EG, followed by exposure to the amyloid peptides Aβ_40_ and Aβ_42_, respectively. The results showed that EG could alleviate the reduction in Aβ_40_-and Aβ_42_-induced cell viability by approximately 8.22 and 8.64%, respectively (Figure 6B).

### Reduction in Aβ-Induced Pro-Inflammatory TNF-α Signaling Through 5-HT_1B_ Pathway by EG

As EG could selectively activate 5-HT_1B_ signaling and alleviate Aβ-induced mortality, we further investigated the underlying mechanisms. Activation of 5-HT_1B_ by its agonist reduces the levels of pro-inflammatory TNF-α in rat nerve cells (Khalilzadeh et al., 2018). The inflammatory cascade is one of the most important processes in Aβ-induced toxicity, and the Aβ oligomer-elevated TNF-α signaling is a cause of memory loss in mice (Lourenco et al., 2013); thus, stimulation of 5-HT_1B_ may relieve Aβ-induced TNF-α signaling. The two amyloid peptides Aβ_42_ and Aβ_40_ induced the death of PC12 cells to a similar extent (Figure 6A). Therefore, we conducted the following experiments with Aβ_42_ because it exhibited neuronal toxicity at a lower concentration than Aβ_40_.

**FIGURE 7:**
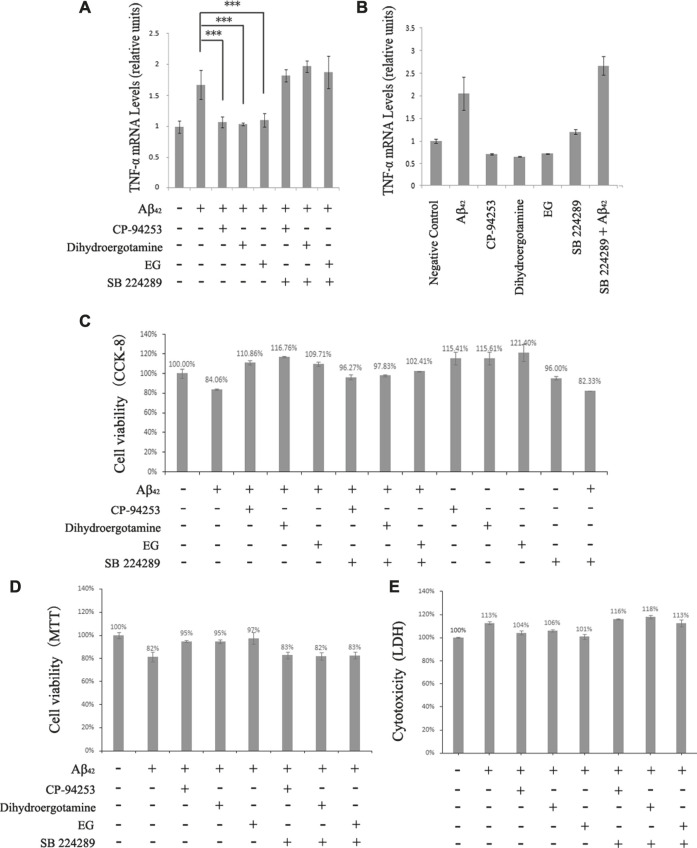
Cell death promoting effect of Aβ_42_ in 5-HT_1B_ over-expressed stable PC12 cells eliminated by TNF-α signaling reduction via 5-HT_1B_ pathway activation. **A**. TNF-α mRNA level induced by Aβ_42_ (20 μM).5-HT_1B_ agonists (CP-94253 and dihydroergotamine) and EG added as indicated at 10^−7^ M. To confirm 5-HT_1B_ mediated pathway role, the 5-HT_1B_ was blocked by its selective antagonist SB 224289 (10^−7^ M), and the TNF-α mRNA level was measured in the presence of dihydroergotamine, CP-94253 and EG. The mean values (±S.D.) of three independent experiments are shown. Asterisks indicate statistical significance (***p<<0.001).** B**. Compounds shown in A were separately tested for their effects on inducing TNF-α expression in 5-HT_1B_ over-expressed, stable PC12 cells.** C**, **D**, **E**. 5-HT_1B_ over-expressed stable PC12 cells were pre-treated with the CP-94253, dihydroergotamine, and EG at 10^−7^ M for 18h, then subjected to Aβ_42_ (20 μM) or not, for 24 h, followed by CCK-8, MTT Kit viability assay and LDH for cytotoxicity. SB 224289 was applied 20 min before CP-94253, dihydroergotamine, and EG when necessary.

To evaluate TNF-α levels under 5-HT_1B_-related conditions, we used quantitative RT-PCR to detect TNF-α mRNA levels in 5-HT_1B_ over-expressing stable PC12 cells (Supplementary Figure S4B) with Aβ_42_ and with or without 5-HT_1B_ activators and blockers (Figure 7A). PC12 cells have endogenous 5-HT_1B_ as well as 5-HT_2A/3/6_ receptor subtypes (Supplementary Figure S4A). We used 5-HT_1B_-over-expressing stable PC12 cells to accentuate the effect of 5-HT_1B_. Consistent with previous studies (Shrewsbury et al., 2008), our results showed that Aβ_42_ elevated TNF-α expression, which could be reversed by treatment with the 5-HT_1B_ agonist CP-94253 and dihydroergotamine and the novel 5-HT_1B_ activator EG. Moreover, the selective serotonin 5-HT_1B_ antagonist SB 224289 blocked EG, CP-94253, and dihydroergotamine-mediated TNF-α restraint under Aβ_42_ conditions. The 5-HT_1B_ activators alone could decrease TNF-α levels in the 5-HT_1B_ over-expressed PC12 cells, whereas the antagonist SB 224289 contributed to pro-inflammatory TNF-α signaling and was synergistic with Aβ_42_ (Figure 7B). These results suggested that EG resorted to the 5-HT_1B_ pathway to reduce Aβ-induced pro-inflammatory TNF-α signaling.

### EG Eliminates the Aβ_42_-Induced Cytotoxic Effect via 5-HT_1B_ Pathway

The Aβ-induced inflammatory response and subsequent neuronal death are considered important events in AD pathology (Hamley, 2012; Unzeta et al., 2016). We found that the death of Aβ_42_-induced 5-HT_1B_ over-expressing stable PC12 cells was significantly reversed by the 5-HT_1B_ agonists and EG, as assessed via the CCK-8 and 3-(4,5-dimethylthiazol-2-yl)-2,5-diphenyltetrazolium bromide (MTT) assays (Figures 7C,D, Supplementary Figure S5A). However, SB 224289 alone did not lead to significant cell death under the indicated conditions (10^−7^ M, 42 h, Supplementary Figure S6). EG and the 5-HT_1B_ agonists reduced the Aβ_42_-induced cytotoxicity that could be blocked by SB 224289 (Figure 7E, Supplementary Figure S5B).

### EG Protection Against Morbidity in *C. Elegans* AD Model

**FIGURE 8:**
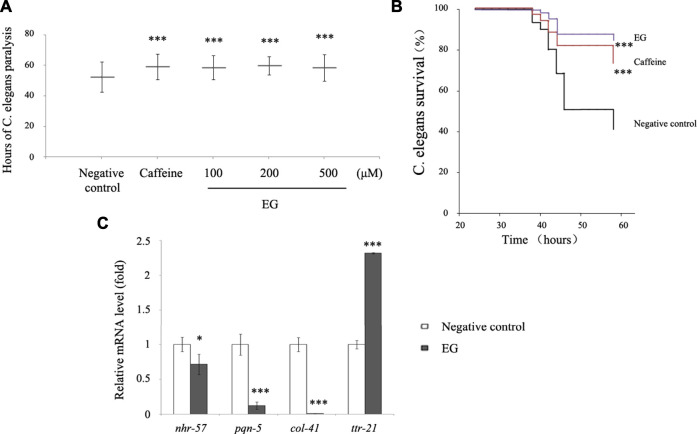
EG retarded transgenic *C. elegans* paralysis in AD model. **(A)**. CL14176 of *C. elegans* strain synchronized to L1 phase, and cultured at 16°C for 24 h, then transferred to 25°C to express amyloid peptides that would result in paralysis. The negative control was untreated (equal volume of water), the positive was exposed to 6.27 mM caffeine, and the EG was measured. The mean paralysis times and the ranks of the individual paralysis times in each group are shown (see Supplementary Table S1). Asterisks indicate statistical significance (***p<<0.001). **(B)** Worms were synchronized and cultured at 16°C for 24 h, then transferred to 25°C for activation. The paralysis phenotypes were detected after 38 h, the number of worms not paralyzed was converted to a percentage, and the “non-paralyzed” percentage was plotted against time activation initiation. The negative control was untreated as described above, the positive was exposed to 6.27 mM caffeine, and the test group to 200 μM EG. Asterisks indicate statistical significance (***p<<0.001). **(C)** Total RNA of the untreated and EG (200 μM) groups was extracted and reverse transcribed to cDNA. The mRNA levels of nhr-57, pqn-5, col-41, and ttr-21 were measured using quantitative RT-PCR. An asterisk indicates statistical significance (*p < 0.05, **p < 0.001, ***p<<0.001). EG: emodin-8-O-β-d-glucopyranoside.

To further explore the ability of EG to afford protection against amyloid peptides, we tested transgenic *C. elegans* for resistance to the induced Aβ expression. The *C. elegans* CL14176 strain (Link et al., 2003), which could be induced to express Aβ by modulating the temperature and leads to paralysis in 2–3 d, was used. This paralysis phenotype could serve as a measurement of Aβ toxicity to assay the effects of compounds protective against Aβ, using the paralysis delay to indicate the suppression of Aβ toxicity in the worm model (Dostal and Link, 2010). The results showed that EG at different concentrations retarded the paralysis time from 6 to 7 h compared with that for the untreated group (∼52 h). The performance of the 200 μM EG group was superior to that of the positive control caffeine group (Supplementary Table S1, Figure 8A). In this model, the survival rate of the EG group at 200 μM was significantly prolonged (Figure 8B). The expression of immune response-related genes that are analogs of the inflammatory effectors in humans, including the nuclear receptor nhr-57, effectors of hypoxia pathway, pqn-5 (prion-like glutamine (Q)/asparagine (N) -5) gene, part of the unfolded protein response pathway that responded to endoplasmic reticulum stress, the collagen gene col-41, and transthyretin-like protein gene ttr-21, between the untreated and EG groups was observed via quantitative RT-PCR (Bellier et al., 2009; Sahu et al., 2012). The EG could significantly reduce the Aβ-induced expression of the immune response-related genes nhr-57, pqn-5, and col-41. However, it failed to suppress ttr-21 expression (Figure 8C), which suggested that EG protected the worm model from AD in a complex manner rather than arbitrarily suppressing all the immune response pathways.

## Discussion

The serotonin receptor 5-HT_1B_, is an important part of the serotonergic system, which regulates crucial processes of the CNS such as cognition, satiety, anxiety, depression, and sleep. 5-HT_1B_ has been considered a drug target for serotonergic system-related disorders (Gadgaard and Jensen, 2020; Monti and Jantos, 2008; Tiger et al., 2018). We identified 5-HT_1B_ as a target of EG, an active ingredient in several Chinese medicinal plants with antidepressant and neuroprotective effects, by using molecular docking, DARTS, and CETSA. Molecular docking provided a structural basis for EG recognition in 5-HT_1B_. Additionally, EG docked into the same 5-HT_1B_ pocket as CP-94253, a 5-HT_1B_ agonist (Figure 1). DARTS allows for the determination of the direct binding of natural products and their extracts to their protein targets. As the conformation and stabilization of the compound-target complex, compared with those of the control protein target, are altered by protease-induced digestion, the compound and target protein interactions can be visualized using western blotting (Chang et al., 2016). The CETSA method can detect the physical interaction between a ligand and target protein in intact cells. The thermodynamic stability of target proteins in drug- or vehicle-treated intact cells varies over changing temperatures, and the target proteins can be monitored using western blotting (Chang et al., 2016). The DARTS results showed that EG could protect the 5-HT_1B_ protein from pronase digestion, compared with 5-HT_1B_ agonists dihydroergotamine (Figures 2A,B) and CP-94253 (Supplementary Figure S2A). In addition, EG promoted thermodynamic stabilization of the 5-HT_1B_ protein compared with dihydroergotamine (Figures 2C–F) and CP-94253 (Supplementary Figure S2B). These methods can also be used to identify other 5-HT_1B_ agonists.

We further studied the structural changes in the 5-HT_1B_ receptor after EG binding using FRET. Intramolecular FRET sensors provide real-time optical evidence for GPCR ligand binding and activation kinetics and can be used as screening tools for molecular ligands toward a particular receptor (Lohse et al., 2008; Xu et al., 2012). We produced an effective 5-HT_1B_ sensor (Figure 4) and detected EG as a ligand with 5-HT_1B_ (Figure 5). However, the changes in FRET signals during treatment with CP-94253 (Figure 4A) and EG (Figure 4B) were in opposite directions, demonstrating that binding of CP-94253 closed the distance between the third intracellular loop and C-terminal of the 5-HT_1B_ receptor, whereas EG extended the distance. Additionally, we found that 5-HT_1B_ could be induced to internalization by agonist CP-94253 and EG (Figure 5), suggesting that EG might be an agonist of 5-HT_1B_. Selective activation of the 5-HT_1B_-mediated ERK1/2 signaling pathway (Figure 6) by EG established its agonist activity toward 5-HT_1B_. Together, the intramolecular FRET 5-HT_1B_ sensor and the subsequent tests of 5-HT_1B_-induced ERK1/2 signaling activation provide a strong evidence that 5-HT_1B_ is a target of and is activated by EG. Further biological functions of EG targeting and 5-HT_1B_ stimulation need to be elucidated.

Overall, activation of central 5-HT_1B_ may have antidepressant (Sanchez et al., 2015), anti-aggression (de Almeida et al., 2001), and antinociceptive effects (Labastida-Ramírez et al., 2020). The therapeutic use of those herbal antidepressants that contain EG has been suggested for the treatment of AD (Jeon et al., 2019; Chen et al., 2020; Li et al., 2020). In this study, the 5-HT_1B_-mediated ERK1/2 phosphorylation by EG and the antifungal (Qi et al., 2005) as well as acetylcholinesterase I (AChE I)- inhibiting (Lin et al., 2015) activities of EG led us to investigate the EG and 5-HT_1B_ interaction in the context of AD. Activation of ERK1/2 signaling is related to the inhibition of Aβ-induced apoptosis (Galvão et al., 2019). Additionally, a series of interesting studies showed that Aβ might act as an antimicrobial peptide, thereby protecting the CNS from infections in mouse models and innate immunity in worms, whereas dysregulated Aβ leads to AD pathology (Kumar et al., 2016). AChE I has been used as a biomarker in AD diagnosis, and AChE I inhibitors have been clinically used for AD treatment (Lin et al., 2015). Both the classic 5-HT_1B_ agonists (CP-94253 and dihydroergotamine) and EG could singly suppress the expression of pro-inflammatory TNF-α and correspondingly improve the survival rates of 5-HT_1B_ over-expressing stable PC12 cells compared with untreated negative control (Figures 7B,C, Supplementary Figure S5A, B). This result might agree with the finding that activated serotonergic pathways promote proliferation (Chilmonczyk et al., 2017). The data in wild-type PC12 cells pointed toward the same trend, although not as significant as those in the 5-HT_1B_ over-expressing stable PC12 cells. EG alleviated Aβ_42_-induced cell death and increased cell viability from approximately 82.71–89.86% (Figure 6B), whereas in 5-HT_1B_ over-expressing stable PC12 cells, the increase was from 84.06 to 109.71% (Figure 7C). In addition, PC12 cells possessed some endogenous serotonin receptor subtypes, including 5-HT_1B_, 5-HT_2A_, 5-HT_3,_ and 5-HT_6_ (Supplementary Figure S4A), that could explain why the wild-type PC12 cells also responded to the classic 5-HT_1B_ agonists (data not shown) and EG in reducing TNF-α levels and were prosurvival. Cell viability in the EG treatment (in Aβ_42_ conditions) was greater than that in the untreated control, indicating the protective effects of EG via the 5-HT_1B_ pathway.

This study provides evidence that 5-HT_1B_ receptor is one of the targets for compounds in Chinese medicinal plant ingredients with cognitive deficit attenuating and antidepressant effects in the treatment of AD. Molecular docking, DARTS, CETSA, FRET, and ERK1/2 phosphorylation tests are all relatively economic, fast, and accurate methods. Further biological functions of the compound targeting 5-HT_1B_ was tested with AD models in cells and worms. However, a variety of herbal compounds are reported to possess cognitive deficit attenuating and antidepressant effects, and the ability of these compounds to target and activate the 5-HT_1B_ still needs to be investigated. Interestingly, caffeine, also a herbal ingredient used as positive control in the worm experiments, was also docked into the 5-HT_1B_ receptor, similar with that of EG, CP94253, and dihydroergotamine (data not shown). These results suggest that caffeine might also be an agonist toward 5-HT_1B_, and support the 5-HT_1B_ agonists’ potential for treatment of AD.

## Data Availability

The original contributions presented in the study are included in the article/Supplementary Material, further inquiries can be directed to the corresponding author.

## Abbreviations

CNS, central nervous system; AD, Alzheimer’s disease; EG, emodin-8-O- β-d-glucopyranoside; DARTS, drug affinity responsive target stability; CETSA, cellular thermal shift assays; FRET, fluorescence resonance energy transfer.

## References

[B1] Bellier A. Chen C. S. Kao C. Y. Cinar H. N. Aroian R. V. (2009). Hypoxia and the Hypoxic Response Pathway Protect against Pore-Forming Toxins in *C. elegans* . Plos Pathog. 5 (12), e1000689. 10.1371/journal.ppat.1000689 20011506 PMC2785477

[B2] Beuming T. Lenselink B. Pala D. McRobb F. Repasky M. Sherman W. (2015). Docking and Virtual Screening Strategies for GPCR Drug Discovery. Methods Mol. Biol. 1335, 251–276. 10.1007/978-1-4939-2914-6_17 26260606

[B3] Chang J. Kim Y. Kwon H. J. (2016). Advances in Identification and Validation of Protein Targets of Natural Products without Chemical Modification. Nat. Prod. Rep. 33 (5), 719–730. 10.1039/c5np00107b 26964663

[B4] Chen S. Y. Gao Y. Sun J. Y. Meng X. L. Yang D. Fan L. H. (2020). Traditional Chinese Medicine: Role in Reducing β-Amyloid, Apoptosis, Autophagy, Neuroinflammation, Oxidative Stress, and Mitochondrial Dysfunction of Alzheimer's Disease. Front. Pharmacol. 11, 497. 10.3389/fphar.2020.00497 32390843 PMC7188934

[B5] Chilmonczyk Z. Bojarski A. J. Pilc A. Sylte I. (2017). Serotonin Transporter and Receptor Ligands with Antidepressant Activity as Neuroprotective and Proapoptotic Agents. Pharmacol. Rep. 69 (3), 469–478. 10.1016/j.pharep.2017.01.011 28324844

[B6] de Almeida R. M. Nikulina E. M. Faccidomo S. Fish E. W. Miczek K. A. (2001). Zolmitriptan--a 5-HT1B/D Agonist, Alcohol, and Aggression in Mice. Psychopharmacology (Berl) 157 (2), 131–141. 10.1007/s002130100778 11594437

[B7] Dostal V. Link C. D. (2010). Assaying β-amyloid Toxicity Using a Transgenic *C. elegans* Model. J. Vis. Exp. 44, 1. 10.3791/2252 PMC3185644 20972410

[B8] Gadgaard C. Jensen A. A. (2020). Functional Characterization of 5-HT1A and 5-HT1B Serotonin Receptor Signaling through G-Protein-Activated Inwardly Rectifying K+ Channels in a Fluorescence-Based Membrane Potential Assay. Biochem. Pharmacol. 175, 113870. 10.1016/j.bcp.2020.113870 32088264

[B9] Galvão F. Jr. Grokoski K. C. da Silva B. B. Lamers M. L. Siqueira I. R. (2019). The Amyloid Precursor Protein (APP) Processing as a Biological Link between Alzheimer's Disease and Cancer. Ageing Res. Rev. 49, 83–91. 10.1016/j.arr.2018.11.007 30500566

[B10] Garcia-Alloza M. Hirst W. D. Chen C. P. Lasheras B. Francis P. T. Ramírez M. J. (2004). Differential Involvement of 5-HT(1B/1D) and 5-HT6 Receptors in Cognitive and Non-cognitive Symptoms in Alzheimer's Disease. Neuropsychopharmacology 29 (2), 410–416. 10.1038/sj.npp.1300330 14571255

[B11] Hamley I. W. (2012). The Amyloid Beta Peptide: A Chemist's Perspective. Role in Alzheimer's and Fibrillization. Chem. Rev. 112 (10), 5147–5192. 10.1021/cr3000994 22813427

[B12] Janoshazi A. Deraet M. Callebert J. Setola V. Guenther S. Saubamea B. (2007). Modified Receptor Internalization upon Coexpression of 5-HT1B Receptor and 5-HT2B Receptors. Mol. Pharmacol. 71 (6), 1463–1474. 10.1124/mol.106.032656 17325130

[B13] Jeon S. G. Song E. J. Lee D. Park J. Nam Y. Kim J. I. (2019). Traditional Oriental Medicines and Alzheimer's Disease. Aging Dis. 10 (2), 307–328. 10.14336/AD.2018.0328 31435482 PMC6667206

[B14] Khalilzadeh M. Panahi G. Rashidian A. Hadian M. R. Abdollahi A. Afshari K. (2018). The Protective Effects of Sumatriptan on Vincristine - Induced Peripheral Neuropathy in a Rat Model. Neurotoxicology 67, 279–286. 10.1016/j.neuro.2018.06.012 29958920

[B15] Koizumi K. Nakajima H. (2014). Serotonin Induces the Migration of PC12 Cells via the Serotonin Receptor 6/cAMP/ERK Pathway. Biomed. Rep. 2 (1), 29–33. 10.3892/br.2013.203 24649064 PMC3917024

[B16] Kontoyianni M. (2017). Docking and Virtual Screening in Drug Discovery. Methods Mol. Biol. 1647, 255–266. 10.1007/978-1-4939-7201-2_18 28809009

[B17] Kumar D. K. Choi S. H. Washicosky K. J. Eimer W. A. Tucker S. Ghofrani J. (2016). Amyloid-β Peptide Protects against Microbial Infection in Mouse and Worm Models of Alzheimer's Disease. Sci. Transl Med. 8 (340), 340ra72. 10.1126/scitranslmed.aaf1059 PMC5505565 27225182

[B18] Labastida-Ramírez A. Rubio-Beltrán E. Haanes K. A. Chan K. Y. Garrelds I. M. Johnson K. W. (2020). Lasmiditan Inhibits Calcitonin Gene-Related Peptide Release in the Rodent Trigeminovascular System. Pain 161 (5), 1092–1099. 10.1097/j.pain.0000000000001801 31977930 PMC7170441

[B19] Ledo J. H. Azevedo E. P. Beckman D. Ribeiro F. C. Santos L. E. Razolli D. S. (2016). Cross Talk between Brain Innate Immunity and Serotonin Signaling Underlies Depressive-like Behavior Induced by Alzheimer's Amyloid-β Oligomers in Mice. J. Neurosci. 36 (48), 12106–12116. 10.1523/JNEUROSCI.1269-16.2016 27903721 PMC6601978

[B20] Li J. M. Zhao Y. Sun Y. Kong L. D. (2020). Potential Effect of Herbal Antidepressants on Cognitive Deficit: Pharmacological Activity and Possible Molecular Mechanism. J. Ethnopharmacol. 257, 112830. 10.1016/j.jep.2020.112830 32259666

[B21] Lin L. Ni B. Lin H. Zhang M. Li X. Yin X. (2015). Traditional Usages, Botany, Phytochemistry, Pharmacology and Toxicology of Polygonum Multiflorum Thunb.: A Review. J. Ethnopharmacol. 159, 158–183. 10.1016/j.jep.2014.11.009 25449462 PMC7127521

[B22] Link C. D. Taft A. Kapulkin V. Duke K. Kim S. Fei Q. (2003). Gene Expression Analysis in a Transgenic *Caenorhabditis elegans* Alzheimer's Disease Model. Neurobiol. Aging 24 (3), 397–413. 10.1016/s0197-4580(02)00224-5 12600716

[B23] Lohse M. J. Nikolaev V. O. Hein P. Hoffmann C. Vilardaga J. P. Bünemann M. (2008). Optical Techniques to Analyze Real-Time Activation and Signaling of G-Protein-Coupled Receptors. Trends Pharmacol. Sci. 29 (3), 159–165. 10.1016/j.tips.2007.12.002 18262662

[B24] Lomenick B. Hao R. Jonai N. Chin R. M. Aghajan M. Warburton S. (2009). Target Identification Using Drug Affinity Responsive Target Stability (DARTS). Proc. Natl. Acad. Sci. U S A. 106 (51), 21984–21989. 10.1073/pnas.0910040106 19995983 PMC2789755

[B25] Lourenco M. V. Clarke J. R. Frozza R. L. Bomfim T. R. Forny-Germano L. Batista A. F. (2013). TNF-α Mediates PKR-dependent Memory Impairment and Brain IRS-1 Inhibition Induced by Alzheimer's β-amyloid Oligomers in Mice and Monkeys. Cell Metab 18 (6), 831–843. 10.1016/j.cmet.2013.11.002 24315369

[B26] Molina D. M. Jafari R. Ignatushchenko M. Seki T. Larsson E. A. Dan C. (2013). Monitoring Drug Target Engagement in Cells and Tissues Using the Cellular Thermal Shift Assay. Science 341 (6141), 84–87. 10.1126/science.1233606 23828940

[B27] Monti J. M. Jantos H. (2008). The Roles of Dopamine and Serotonin, and of Their Receptors, in Regulating Sleep and Waking. Prog. Brain Res. 172, 625–646. 10.1016/S0079-6123(08)00929-1 18772053

[B28] Qi H. Y. Zhang C. F. Wang Z. T. Zhang M. (2005). *Studies On Constituents and Antifungal Activity of Polygonum Cillinerve* Chinese. Pharm. J. 40, 819–822.

[B29] Sahu S. N. Lewis J. Patel I. Bozdag S. Lee J. H. LeClerc J. E. (2012). Genomic Analysis of Immune Response against *Vibrio cholerae* Hemolysin in *Caenorhabditis elegans* . Plos One 7 (5), e38200. 10.1371/journal.pone.0038200 22675448 PMC3364981

[B30] Sanchez C. Asin K. E. Artigas F. (2015). Vortioxetine, a Novel Antidepressant with Multimodal Activity: Review of Preclinical and Clinical Data. Pharmacol. Ther. 145, 43–57. 10.1016/j.pharmthera.2014.07.001 25016186

[B31] Shrewsbury S. B. Cook R. O. Taylor G. Edwards C. Ramadan N. M. (2008). Safety and Pharmacokinetics of Dihydroergotamine Mesylate Administered via a Novel (Tempo) Inhaler. Headache 48 (3), 355–367. 10.1111/j.1526-4610.2007.01006.x 18179563

[B32] Tiger M. Varnäs K. Okubo Y. Lundberg J. (2018). The 5-HT1B Receptor - a Potential Target for Antidepressant Treatment. Psychopharmacology (Berl) 235 (5), 1317–1334. 10.1007/s00213-018-4872-1 29546551 PMC5919989

[B33] Unzeta M. Esteban G. Bolea I. Fogel W. A. Ramsay R. R. Youdim M. B. (2016). Multi-Target Directed Donepezil-like Ligands for Alzheimer's Disease. Front. Neurosci. 10, 205. 10.3389/fnins.2016.00205 27252617 PMC4879129

[B34] Ward R. J. Alvarez-Curto E. Milligan G. (2011). Using the Flp-In™ T-Rex™ System to Regulate GPCR Expression. Methods Mol. Biol. 746, 21–37. 10.1007/978-1-61779-126-0_2 21607850

[B35] Xu T. R. Ward R. J. Pediani J. D. Milligan G. (2012). Intramolecular Fluorescence Resonance Energy Transfer (FRET) Sensors of the Orexin OX1 and OX2 Receptors Identify Slow Kinetics of Agonist Activation. J. Biol. Chem. 287 (18), 14937–14949. 10.1074/jbc.M111.334300 22389503 PMC3340242

