# Supplementary material for: A Novel 5-HT1B Receptor Agonist of Herbal Compounds and One of the Therapeutic Uses for Alzheimer’s Disease
Source: Front Pharmacol. 2021 Sep 6;12:735876. doi: 10.3389/fphar.2021.735876 (PMC8450432; doi:10.3389/fphar.2021.735876)
Supplement: Supplementary file 1 [file DataSheet1.docx]

**Supplemental materials**


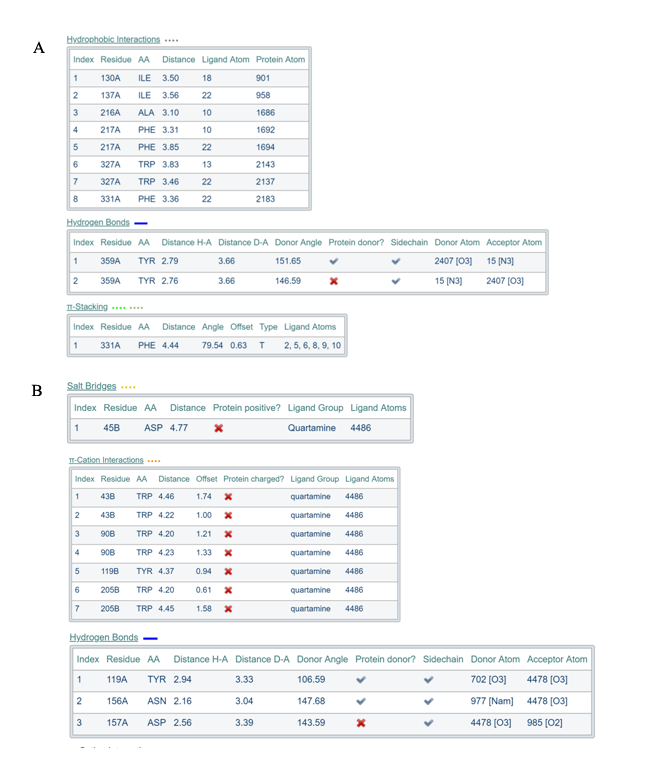


Supplemental Figure 1. Quantitative results for CP-94253 (A) and EG (B) docking into 5-HT_1B_

5-HT_1B_: serotonin 1B receptor


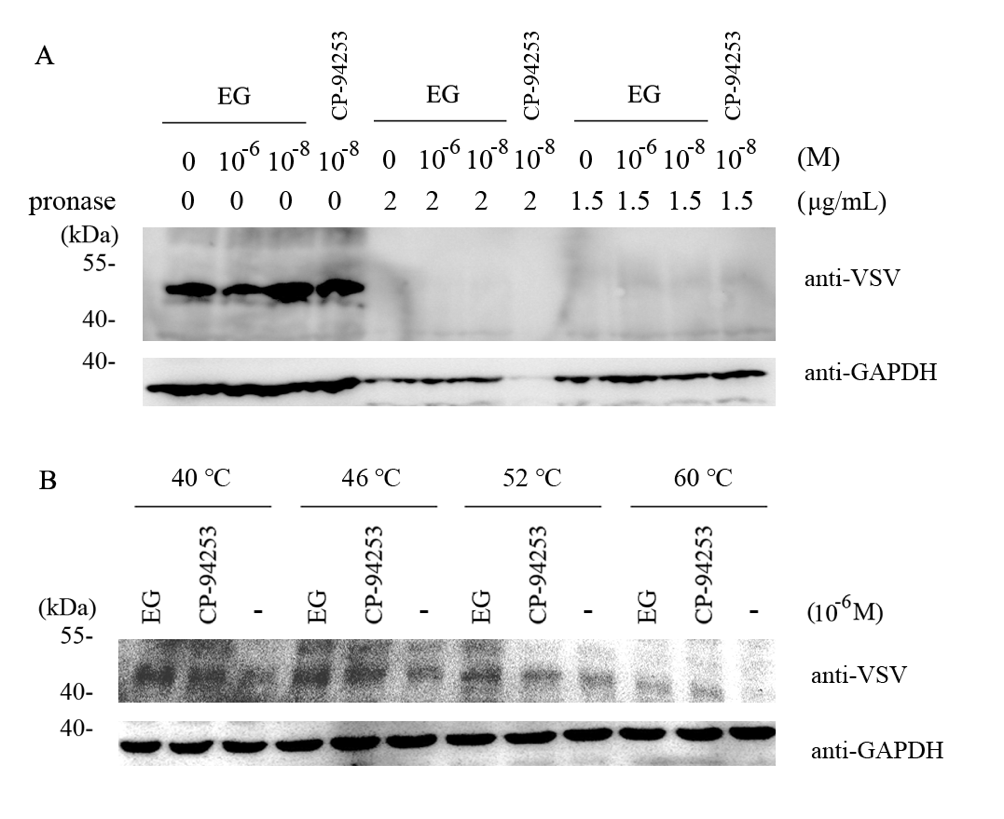


Supplemental Figure 2. EG interaction with 5-HT_1B_ compared with CP-94253 in DARTS and CETSA experiments

DARTS: drug affinity responsive target stability; CETSA: cellular thermal shift assay

GAPHD: glyceraldehyde 3-phosphate dehydrogenase; VSV: vesicular stomatitis virus; EG: emodin-8-O-β-D-glucopyranoside; 5-HT_1B_: serotonin 1B receptor


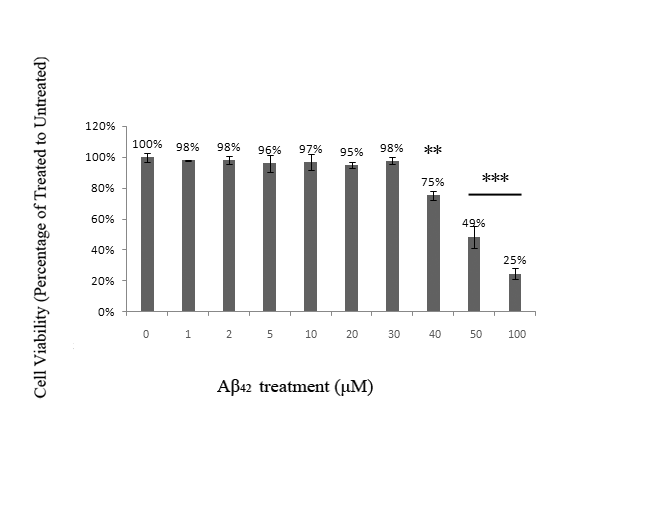


Supplemental Figure 3. Aβ_42_ peptide-induced neuronal death

SH-SY5Y cells were treated with amyloid peptides Aβ_42_ for 24 h, at gradient concentrations from 1 to 100 μM, followed by Cell Counting Kit-8 (CCK-8) viability assay.


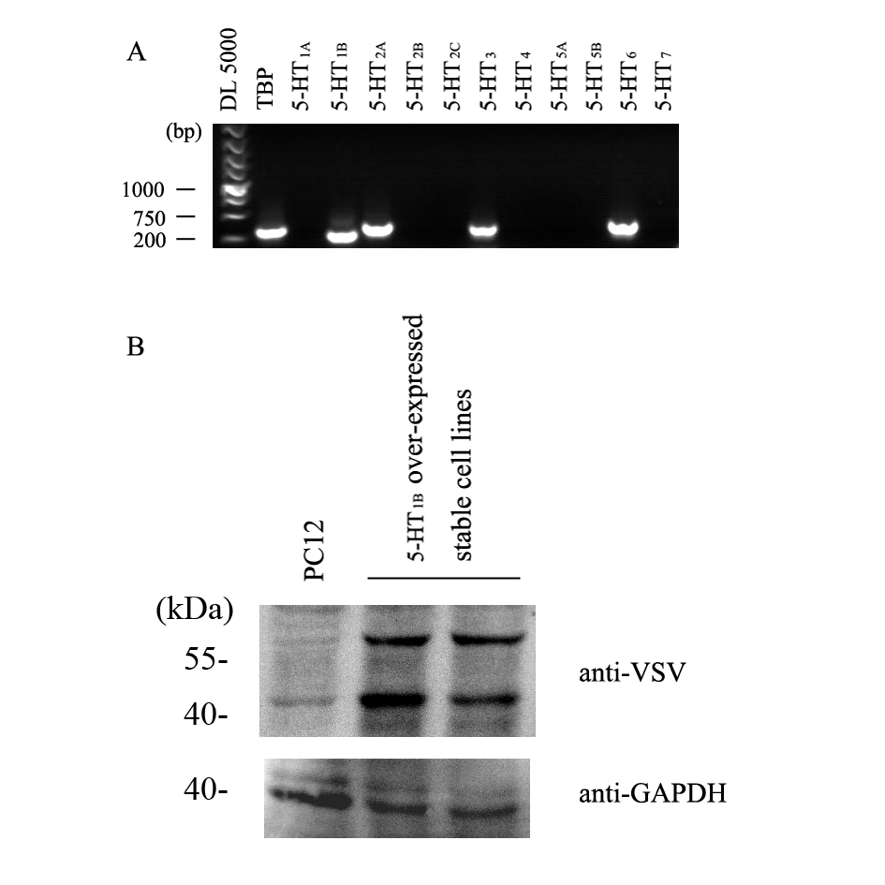


Supplemental Figure 4. Establishment of 5-HT_1B_ over-expressed stable PC12 cell lines

A. PC12 cells were subjected to total RNA extraction and RT-PCR to detect endogenous 5-HT receptor subtypes. TATA-binding protein was positive control. Primers were previously described [[35](#_ENREF_35)].

B. PC12 cells were transfected with VSV-G-mGluR5-5-HT_1B_ construct, added to 100 ng.mL^-1^ hygromycin to select for stably transfected cells.

GAPHD: glyceraldehyde 3-phosphate dehydrogenase; VSV: vesicular stomatitis virus; EG: emodin-8-O-β-D-glucopyranoside; 5-HT_1B_: serotonin 1B receptor


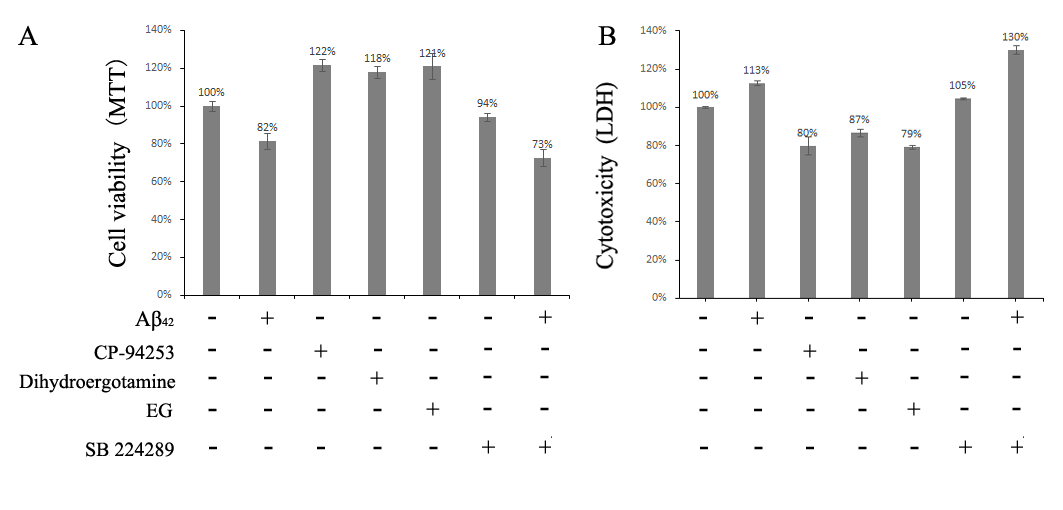


Supplemental Figure 5. 5-HT_1B_ over-expressed stable PC12 cells pre-treated with-94253, dihydroergotamine, and EG at 10^-7^ M for 18 h, then subjected to 20 μM Aβ_42_ or no, for 24 h, followed by MTT Kit assay for viability and LDH for cytotoxicity.

MTT: 3-(4,5-dimethylthiazol-2-yl)-2,5-diphenyltetrazolium bromide; LDH: lactate dehydrogenase; 5-HT_1B_: serotonin 1B receptor; EG: emodin-8-O-β-D-glucopyranoside; CCK-8: Cell Counting Kit-8; MTT: 3-(4,5-dimethylthiazol-2-yl)-2,5-diphenyltetrazolium bromide


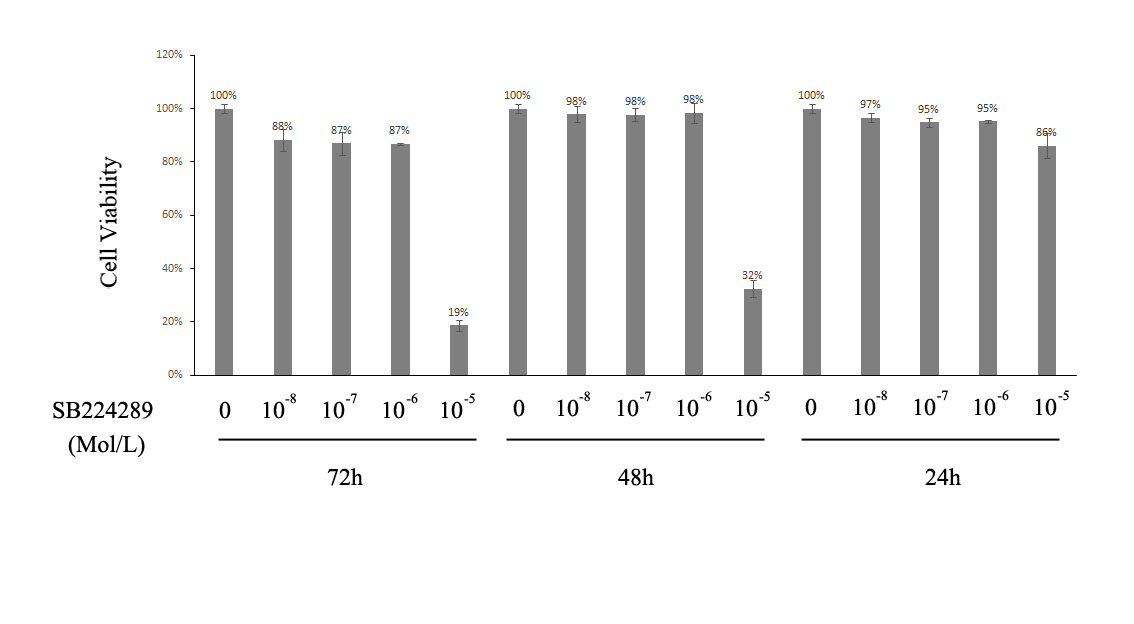


Supplemental Figure 6. SB 224289-induced neuronal death

PC12 cells were treated with 5-HT_1B_ selective antagonist SB 224289 for 24 to 72 h, at gradient concentrations from 10^-8^ to 10^-5^ M, followed by Cell Counting Kit-8 (CCK-8) viability assay.

Supplemental Table 1 Paralysis in C. elegans strain CL4176 worms exposed to indicated compounds

| Sample | |  | N (total) | SE of mean (Hours) | *P* value of log-rank |
| --- | --- | --- | --- | --- | --- |
| Negative control | |  | 166 | 52.37349±0.77749 |  |
| caffeine | |  | 173 | 59.12139±0.63022 | *P*<0.001 |
| EG | 100 μM |  | 180 | 58.56667±0.57407 | *P*<0.001 |
|  | 200 μM |  | 232 | 59.75±0.39885 | *P*<0.001 |
|  | 500 μM |  | 234 | 58.3504±0.5627 | *P*<0.001 |
